# Clinical presentation, acute care management and discharge information of patients with thoracic trauma in South Africa and Sweden: a prospective multicenter observational study

**DOI:** 10.1007/s00068-024-02753-y

**Published:** 2025-01-16

**Authors:** Heleen van Aswegen, Ronel Roos, Anna Svensson-Raskh, Annie Svensson, Maria Sehlin, Eva-Corina Caragounis, Frank Plani, Monika Fagevik-Olsén

**Affiliations:** 1 Physiotherapy, Faculty of Health Sciences, University of the Witwatersrand, Johannesburg, South Africa; 2 Division of Physiotherapy, Karolinska Institutet, Stockholm, Sweden; 3 Physiotherapy, Karolinska University Hospital, Stockholm, Sweden; 4 Department of Community Medicine and Rehabilitation, Umeå University, Umeå, Sweden; 5 Department of Surgery, Sahlgrenska University Hospital, Gothenburg, Sweden; 6 Sahlgrenska Academy, University of Gothenburg, Gothenburg, Sweden; 7 Trauma Division, Netcare Alberton Hospital, Johannesburg, South Africa; 8 Department of Physical Therapy, Sahlgrenska University Hospital, Gothenburg, Sweden

**Keywords:** Thoracic trauma, Pain, Shortness of breath, Pulmonary complications, Length of stay

## Abstract

**Purpose:**

Thoracic trauma causes pain and hospitalisation. Middle- and high-income countries have different trauma contexts and populations. To report patients’ clinical presentation (pain and shortness of breath) and its influence on hospital length of stay (LOS), acute care management, and discharge destinations in South Africa (SA) and Sweden.

**Methods:**

Prospective observational multicenter study by means of clinical record review. Two centers in SA and four centers in Sweden participated. One thousand nine hundred and eighteen adults with thoracic trauma were screened over the 20 months period. Study objectives guided information retrieved from clinical records. Statistical analysis was done with significance at p-value < 0.05.

**Results:**

Three-hundred-sixty-four participants were recruited with most being male (n = 170/179 (95%) SA; n = 125/185 (68%) Sweden). Type and mechanism of injury differed (SA penetrating (82%) versus Sweden blunt (95%); SA assaults (90%) versus Sweden falls (44%)). Unilateral haemopneumothorax was common (SA 68%, Sweden 35%) and managed with intercostal drainage. Rib cage injuries were common in the Swedish cohort with rib fixation surgery for 17%. Physiotherapy treatment frequency was mostly daily. Blunt injury resulted in higher pain levels during deep breathing (day 1: p = 0.014; day 2: p < 0.001; day 3: p < 0.001) and shortness of breath during activity (day 1: p = 0.036; day 2: p = 0.003; day 3: p < 0.001). LOS was shorter for SA cohort (5 (± 4) versus 7 (± 5) days; p = 0.024). Age influenced LOS in the blunt injury group. Discharge destination was mostly home (99% SA, 56% Sweden).

**Conclusion:**

Priority care is indicated for those who are older and have blunt thoracic injury to prevent pulmonary complications and prolonged hospitalisation.

**Supplementary Information:**

The online version contains supplementary material available at 10.1007/s00068-024-02753-y.

## Introduction

Traumatic injury continues to be a significant health risk and cause of death and disability on a global level [1]. Thoracic trauma is observed in approximately two-thirds of patients with traumatic injury and is the third most common cause of morbidity and mortality among such patients [2, 3]. The incidence of traumatic injury differs according to region and a country’s economic standing [4, 5]. In South Africa (SA), injuries contribute significantly to the quadruple burden of disease with an incidence of 100.3 per 100 000 population [6]. The incidence of road traffic accidents has decreased by 29% between 2009 and 2017 [6]; however penetrating injuries to the thorax are rising with 82%−94% due to stab wounds and 6%−18% due to gunshot wounds [7]. In Sweden, trauma is the sixth leading cause of death in all age groups according to the Swedish Trauma Registry [8]. Blunt trauma is the dominating injury mechanism, occurring in approximately 90% of cases, although penetrating injuries are on the rise [8]. Road traffic accidents make up about 50% of all trauma cases in Sweden [8]. In both countries, males are reported to be more involved in thoracic trauma than their female counterparts [6, 8, 9].

Severity of thoracic injury determines the need for hospitalisation. Most patients can be managed conservatively through multidisciplinary team involvement, with only 10% requiring surgical intervention [10]. Injury to the thorax causes pain from structural and tissue damage which presents as nociceptive and/or neuropathic in nature [11]. Implementation of appropriate pain relief strategies is essential during patient management [12–14]. Persistent pain leads to reflex shortness of breath and, if either is not well-managed, increases patients’ risk for pulmonary complications such as atelectasis, pneumonia, respiratory distress, and respiratory failure [3, 12, 13]. In addition, injury severity, age and prior health condition of the patient influences their risk of developing such complications [12]. Pulmonary complications may necessitate admission to an intensive care unit and result in prolonged hospital length of stay (LOS) [15].

No prospective data, to mirror actual clinical practice, exists on the levels of pain and shortness of breath that patients with thoracic trauma experience during their hospital stay, acute care management provided to such patients from countries with varying trauma contexts and populations, pulmonary complications developed, and discharge information. This paper reports on these outcomes and identifies factors that influenced patients’ hospital LOS.

## Methods

### Study design and population

A prospective multicenter observational study by means of a clinical record review of adult patients with thoracic trauma needing hospitalization, was conducted. Thoracic trauma was defined as blunt or penetrating injury resulting in pneumothorax, haemothorax, fractures of the chest wall, diaphragm and/or lung laceration, and/or pulmonary contusion. The STROBE checklist was used in reporting of this study.

All six participating sites were public healthcare sector university-affiliated hospitals. In SA, Charlotte Maxeke Johannesburg Academic and Chris Hani Baragwanath Academic hospitals, situated in Johannesburg, participated. Ethics approval was obtained from University of the Witwatersrand Human Research Ethics (Medical) committee (Date: 27/06/2020, No. M200222) for both study sites. Permissions and consent were obtained from the National Department of Health, and the chief executive officers, trauma unit managers, physiotherapy heads-of-department and patients at these two participating sites. In Sweden, Sahlgrenska University hospital (Gothenburg), Karolinska University hospital (Stockholm), University hospital of Umeå (Umeå), and Skåne University hospital (Lund) participated. Ethics approval was obtained from the Swedish Ethical Review Authority for the region of Västra Götaland and all study-sites were included in the approval (Date: 22/10/2019, No. Dnr2019-04848). Consent was obtained from all head-of-departments and the patients at the four participating sites.

The South African patients approached for participation were managed in the trauma ICU and wards of the two participating hospitals. The Swedish patients were managed either in trauma centres (Karolinska and Sahlgrenska University hospitals) or surgical wards with trauma profile (Umeå and Skåne University hospitals). Participants were treated according to standard trauma and physiotherapy practice at all hospitals. Physiotherapy services were always available on weekdays in all hospitals, but the availability of physiotherapists on duty during weekends varied. New patient admissions or patients with acute respiratory symptoms at weekends could receive physiotherapy services at Chris Hani Baragwanath Academic hospital, and Karolinska and Sahlgrenska University hospitals while treatment at the other hospitals were conducted individually or supervised by nursing staff, with standard service delivery resuming the following Monday.

### Inclusion and exclusion criteria

Patients with thoracic trauma who were 18 years or older and of both sexes were consecutively screened at the ICUs and trauma or surgical wards of all participating sites for possible inclusion, using the ICU or ward admission registers. Those with dementia, acute or previously diagnosed spinal cord injury, moderate (Glasgow coma scale (GCS) 9–12) and severe (GCS < 9) traumatic brain injury, complex pelvic fractures, lower limb fractures, lower limb amputation that restricted active mobilisation, and extensive abdominal trauma (e.g. repeat laparotomy procedure or open abdomen with drainage system) were excluded.

### Data collected

Meetings were held with the clinical physiotherapy staff who worked in the trauma units and wards of the participating study sites to explain the study purposes. The physiotherapists that oversee the trauma ICU, high care units and surgical/trauma wards at the participating sites were responsible for participant recruitment, as they screened the wards and units daily for new admissions. Participants gave their written consent before any of their data were captured. The physiotherapists captured participant details on study specific data forms. Information captured included demographics (age, sex, height, weight, smoking history, presence of chronic pulmonary disease), clinical presentation (type and mechanism of injury, pain, shortness of breath), medical, surgical and physiotherapy management received, pulmonary complications developed during hospitalisation, and discharge information (length of stay, and discharge destination). All participating hospitals in SA and Sweden had the same protocol for data capturing and included the data prospectively. Variables covering days before participant consent were not registered.

**Fig. 1:**
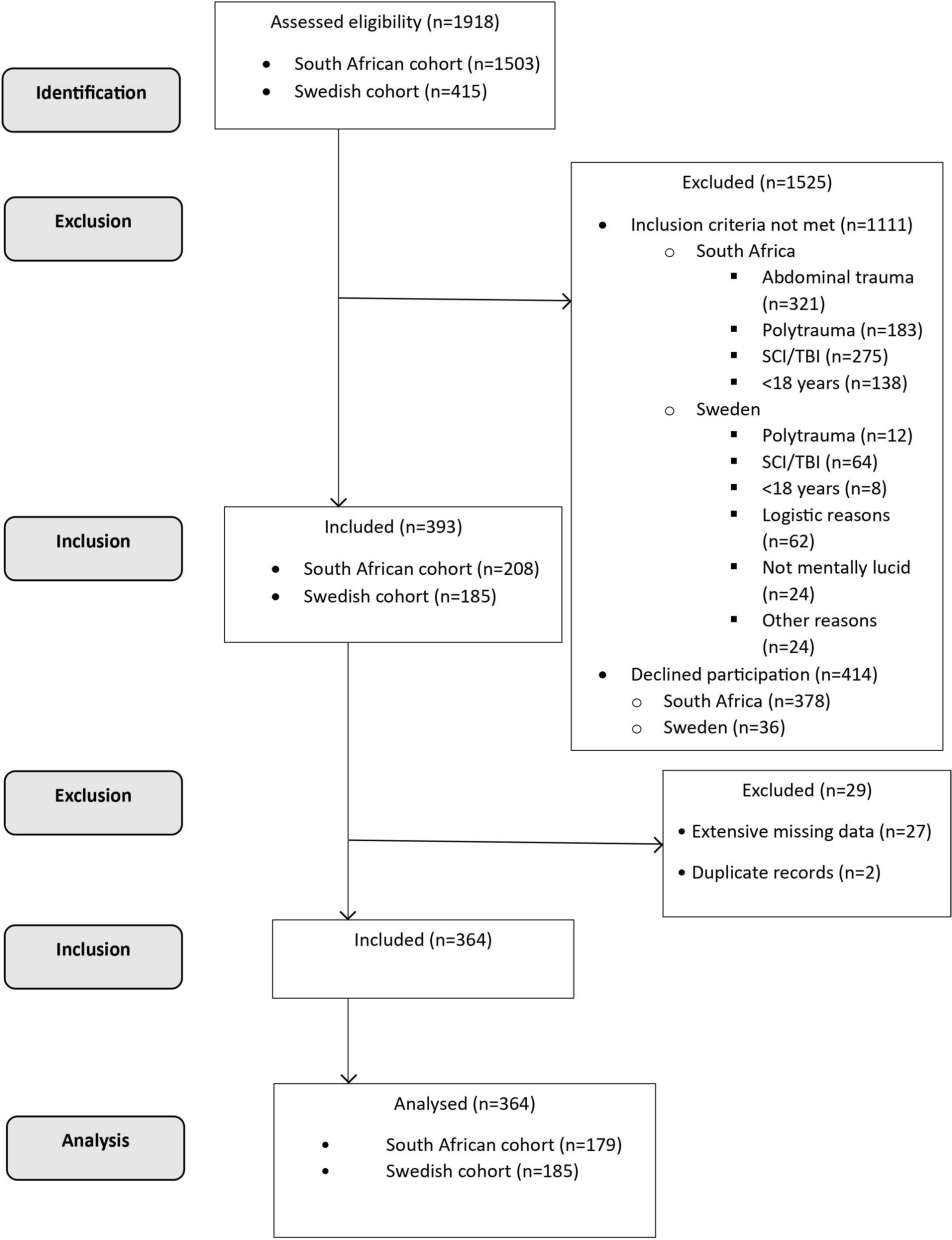
Participant recruitment and flow through the study

Recruitment and data collection occurred over a total of 20 months at the SA participating sites (October 2020 to October 2021 (Chris Hani Baragwanath Academic hospital); August 2022 to February 2023 (Charlotte Maxeke Johannesburg Academic hospital)) and over 13 months in Sweden (September 2021 to September 2022). Figure 1 summarises the process of recruitment of participants. Data were captured onto two electronic databases (Research Electronic Data Capture hosted at the University of the Witwatersrand (South Africa) and Google Drive (Sweden)).

### Statistical analysis

Data were cleaned and imported to Statistical Package for Social Sciences (SPSS) Version 29.0 (IBM® Corporation, New York, USA) for analysis. Descriptive statistics were used to summarise the data. Comparison of findings between cohorts were made using Pearson Chi-squared test (categorical variables e.g. types and mechanisms of injury), Mann Whitney-U test (ordinal variables e.g. Numeric Rating Scale (NRS) for pain and Borg (CR-10) scale for shortness of breath), and independent t-test (continuous variables e.g. age and length of stay). Significance was determined at p < 0.05. Univariate linear regression was done to identify contributing factors to hospital LOS. The dependent variable was logLOS as the LOS data were skewed. The independent variables were age, type of injury (blunt vs penetrating), rib fractures (yes/no), pulmonary complications (yes/no), other injuries (yes/no), moderate-to-severe pain (≥ 4/10) reported during deep breathing on days 1–3 (yes/no), and lastly moderate-to-severe pain (≥ 4/10) reported during deep breathing on day 3 (yes/no). The independent variables that had a significant impact on LOS at univariate analysis were entered into a backward linear regression for multivariate analysis to determine which impacted on LOS.

## Results

### Patient characteristics and types and mechanisms of injury

A total of 1 918 patients were admitted to the participating sites during the study period (Fig. 1). Three-hundred-sixty-four patients with thoracic trauma participated of which 179 (49%) were from South Africa and 185 (51%) from Sweden. Characteristics of the participants are summarized in Table 1.

**Table 1:** Characteristics of the patients with thoracic trauma in the study cohorts

Variable	Description	South African cohort (n = 179)	Swedish cohort (n = 185)	p-value between cohorts
Sex (n,%)	Male	170 (95)	125 (68)	< 0.001
	Female	9 (5)	60 (32)	
Age (mean, SD)	Years	33 (10)	58 (18)	< 0.001
BMI (mean, SD)	kg/m^2^	23 (5)	26 (6)	< 0.001
Current smokers (n,%)		96 (54)	24 (13)	< 0.001
Pulmonary disease (n,%)		1 (1)	20 (11)	< 0.001

*BMI* body mass index

Participants were mostly male. Most were between the ages of 30 to 64 years (n = 103 (58%) SA; n = 107 (58%) Sweden). The SA cohort was significantly younger than the Swedish cohort. More than half of the SA cohort were current smokers, and 21% (n = 37) had a BMI of 25–30 kg/m^2^ compared to the Swedish cohort of whom 13% were current smokers and 55% (n = 84) had a raised BMI. More Swedish participants had chronic respiratory disease prior to thoracic trauma.

All participants received a computed tomography scan on admission to the emergency departments. Most were recruited from a ward setting (SA 92%, Sweden 69%) with few recruited within ICU (SA 6%, Sweden 3%). The types and mechanisms of thoracic injury sustained are summarized in Table 2.

**Table 2:** Types and mechanisms of thoracic injury sustained

Variable (n, %)	Description	South African cohort (n = 179)	Swedish cohort (n = 185)	p-value between cohorts
Type of injury	Blunt injury	33 (18)	175 (95)	< 0.001
	Penetrating injury	146 (82)	10 (5)	
Mechanism of injury	Assault	161 (90)	18 (10)	< 0.001
	Attempted suicide	1 (1)	4 (2)	
	MVC	9 (5)	31 (17)	
	PVC	5 (3)	30 (16)	
	Cycling accident	0	7 (4)	
	Falls	0	81 (44)	
	Miscellaneous	3 (2)	14 (8)	
Rib fractures	1–3 ribs fractured unilaterally	15 (8)	61 (33)	0.005
	4–6 ribs fractured unilaterally	12 (7)	42 (23)	
	> 3 ribs fractured bilaterally	2 (1)	29 (16)	
	Flail rib injury	0	40 (22)	
Intrapleural abnormality	Pneumothorax unilateral	41 (23)	41 (22)	0.023
	Pneumothorax bilateral	0	2 (1)	
	Haemo-/haemopneumothorax unilateral	122 (68)	64 (35)	
	Haemo-/haemopneumothorax bilateral	10 (6)	4 (2)	
Pulmonary contusion	Yes	9 (5)	42 (23)	< 0.001
Lung laceration	Yes	6 (3)	8 (4)	0.787
Diaphragm rupture	Yes	4 (2)	2 (1)	0.445
Sternal fracture	Yes	0	16 (9)	< 0.001

*MVC* motor vehicle crash, *PVC* pedestrian vehicle crash

**Fig. 2:**
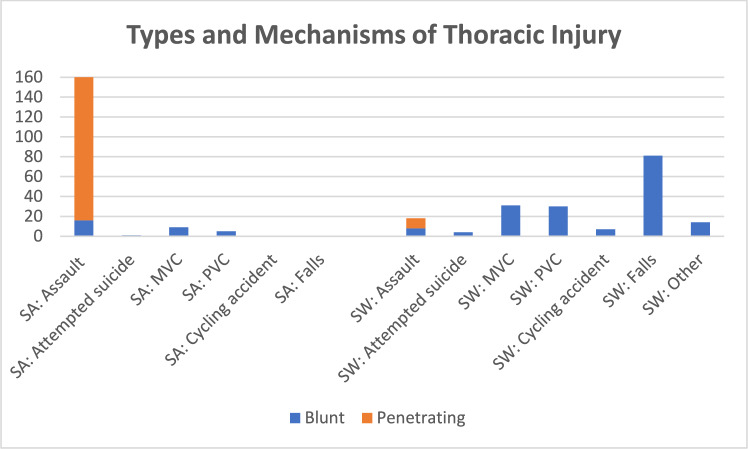
Type and mechanism of thoracic injury per cohort. *SA* South Africa, *SW* Sweden, *MVC* motor vehicle crash, *PVC* pedestrian vehicle crash

The type of injury differed as most SA participants had penetrating thoracic injury (82%) and Swedish participants had blunt injury (95%). Mechanism of injury was different between the two cohorts with the SA cohort injured through assault (90%) compared to the Swedish cohort who were injured through falls (44%) (Fig. 2).

Rib fractures and flail rib injury were higher in the Swedish cohort with no flail rib injury reported for the SA cohort. Most participants in both cohorts sustained unilateral haemopneumothorax. The Swedish cohort had significantly more other orthopaedic injuries (Supplementary Table 1). There was no difference in the types of internal organ injuries sustained by both cohorts.

### Acute care management

Patients with thoracic trauma mostly received conservative management and physiotherapy with some needing surgical interventions. Table 3 summarizes and compares the conservative and surgical interventions used within the two cohorts during acute care management.

**Table 3:** Conservative and surgical management provided to patients with thoracic trauma during their hospitalization

Acute care management (n,%)	South African cohort (n = 179)	Swedish cohort (n = 185)	p-value between cohorts
Analgesia	176 (98)	185 (100)	0.148
Sedation	6 (3)	7 (4)	0.825
Mechanical ventilation	7 (4)	4 (2)	0.339
Non-invasive ventilation	0	3 (2)	0.087
High flow nasal oxygen therapy	3 (2)	3 (2)	0.973
Oxygen therapy	86 (48)	96 (52)	0.023
Intercostal drain insertion	165 (92)	69 (37)	< 0.001
Rib fixation surgery	0	32 (17)	< 0.001
Other surgeries	24 (13)	29 (16)	0.484

**Fig. 3:**
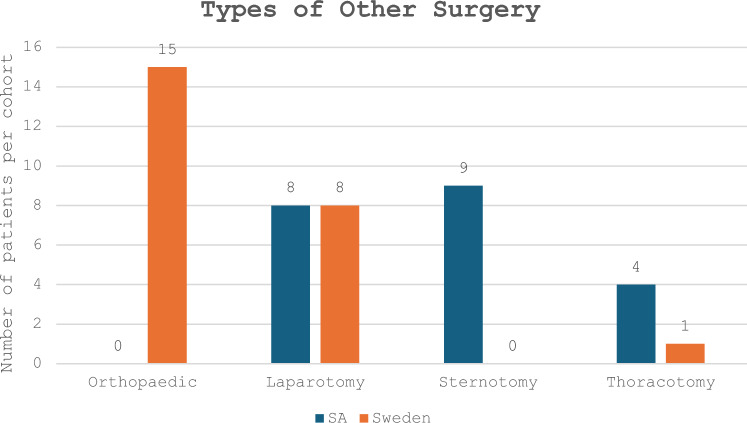
Number of patients who received other types of surgery presented per cohort

The management provided to both cohorts was similar as most participants received analgesia (intravenous or oral opioid therapy) and few were sedated, but more SA participants (52%) were breathing at room air than their Swedish counterparts (34%) (p = 0.023). More SA participants (92%) were managed with intercostal drainage systems than their Swedish counterparts (37%) (p < 0.001) because of a higher incidence of penetrating trauma. Rib fixation surgery was performed for 17% of the Swedish cohort and none of the SA cohort. The types of other surgeries performed in the two cohorts are summarized in Fig. 3.

Significantly more participants in the SA cohort (n = 160, 93%) received physiotherapy management from Day 1 after admission to the hospital than in the Swedish cohort (n = 106, 73%, p < 0.001). Most SA participants (99%) received one physiotherapy treatment session per day over the first three days of hospital stay, whereas some participants in the Swedish cohort (7%−9%) received two or more treatment sessions. Details of physiotherapy management approaches used for patient care in the two cohorts are reported elsewhere.

### Blunt vs penetrating thoracic injury

Participants with blunt thoracic injury made up 57% of the SA and Swedish cohorts combined (n = 208/364). Table 4 summarizes and compares the demographic and clinical data for the blunt and penetrating thoracic injury groups.

**Table 4:** Demographic and clinical information for participants with blunt thoracic injury compared to those with penetrating injury

Variables	Description	Blunt trauma group (n = 208)	Penetrating trauma group (n = 156)	p-value
Age (mean, SD)	Years	56 (18)	32 (9)	< 0.001
Age (mean, SD) *per country* SA Sweden	Years	39 (12) 60 (17)	32 (9) 34 (14)	Blunt trauma group: < 0.001 Penetrating trauma group: 0.57
Chronic respiratory disease prior to injury (n, %)	Yes	20 (10)	1 (1)	–
Pulmonary complications developed (n, %)	Yes	24 (12)	5 (3)	0.004

Participants who presented with penetrating thoracic injury were significantly younger than those with blunt injury. South African participants with blunt thoracic injury were younger than those from Sweden. More participants with blunt trauma had chronic respiratory disease prior to injury with significantly more incidences of pulmonary complications compared to those with penetrating injury.

### Pain and shortness of breath experienced and pulmonary complications

**Fig. 4:**
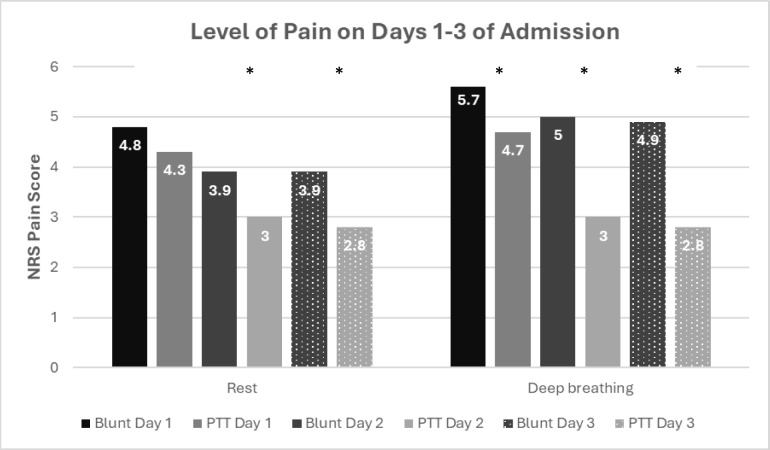
Level of pain reported by participants with blunt injury compared to those with penetrating injury on days 1–3 of admission at rest and during deep breathing. *Significant difference at p < 0.05. *NRS* Numeric Rating Scale, *Blunt* blunt thoracic trauma, *PTT* penetrating thoracic trauma

Level of pain experienced at rest and during deep breathing was measured with the NRS (Fig. 4).

The blunt injury group had significantly more pain at rest on days 2 (p = 0.018) and 3 (p = 0.036) compared to the penetrating injury group. During deep breathing on days 1–3 (day 1: p = 0.014; day 2: p < 0.001; day 3: p < 0.001) the blunt injury group reported significantly higher levels of pain. Subgroup analysis was performed to distinguish between the pain reported during rest and deep breathing by SA and Swedish participants with blunt thoracic trauma and those with penetrating trauma. No significant differences were found in level of pain reported over the first three days of hospitalisation. Moderate-severe pain (> 4/10 NRS) during deep breathing on days 1–3 was reported by more participants with blunt injury (n = 133; 83%) than those with penetrating injury (n = 85; 61%; p < 0.001). On day 3 72% of those with blunt trauma still reported moderate-severe pain on deep breathing compared to 33% with penetrating thoracic trauma.

**Fig. 5:**
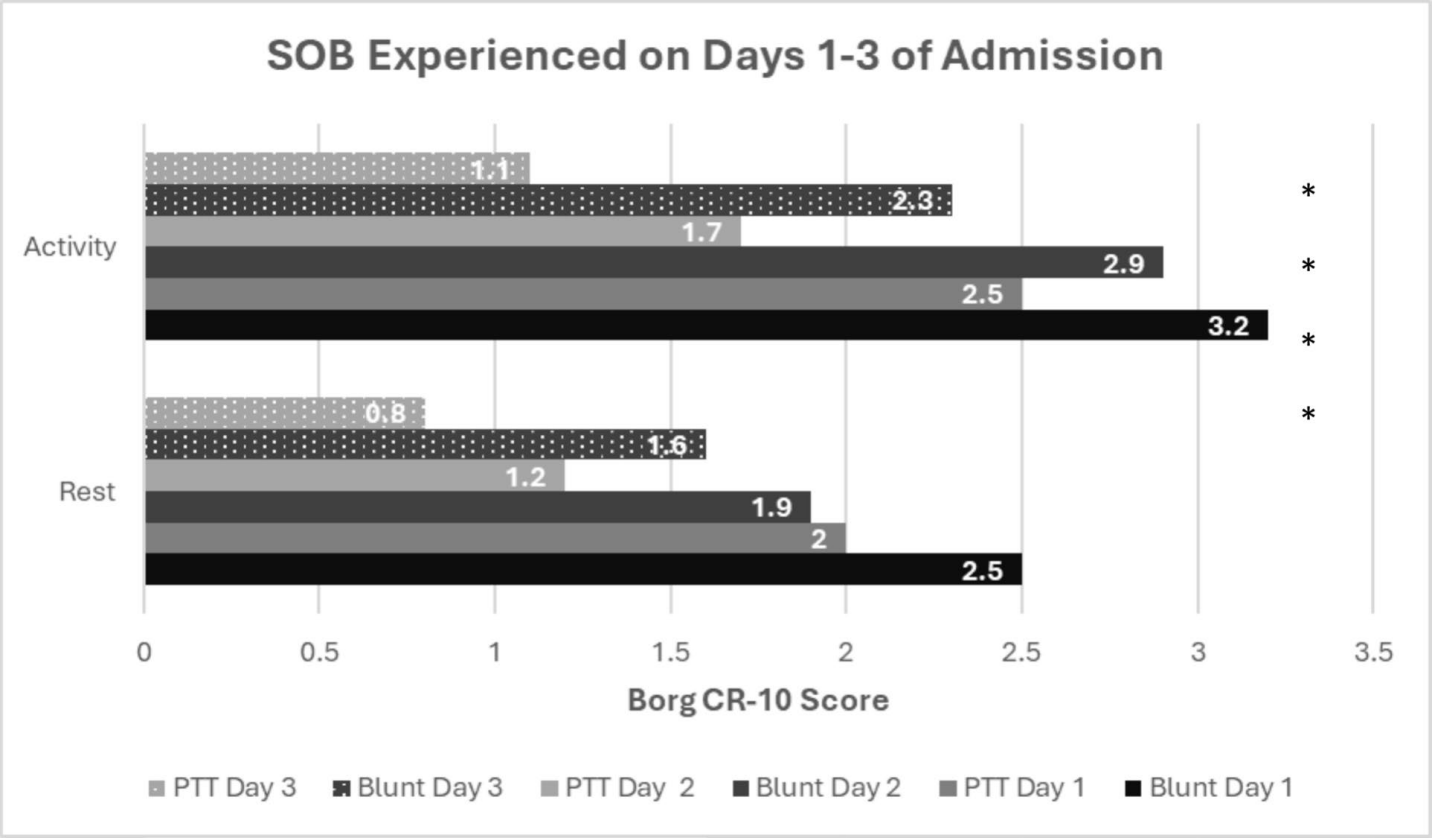
Shortness of breath reported by participants at rest and during activity on the first three days of admission. *Significant difference at < 0.05

Shortness of breath at rest and during activity was reported using the Borg CR-10 scale (Fig. 5).

The blunt thoracic injury group had significantly more shortness of breath at rest on day 3 (p = 0.006) and when performing activity on days 1–3 (day 1: p = 0.036; day 2: p = 0.003; day 3: p < 0.001). Subgroup analysis showed that the Swedish cohort with blunt injury (n = 52) experienced more shortness of breath at rest on day 3 (p = 0.028) than the SA cohort (n = 11) with blunt injury. No differences in shortness of breath during activity were found between these cohorts with blunt injury. Subgroup analysis for those with penetrating injury was not possible due to missing data.

Pulmonary complications were rare. More participants with blunt injury (n = 24, 12%) developed complications than those with penetrating injury (n = 5, 3%; p = 0.004). Atelectasis was the main type of complication and occurred in 15 participants with blunt trauma and five with penetrating trauma. Eight participants with blunt trauma developed pneumonia and a further two developed respiratory failure and needed intubation and mechanical ventilation. These latter complications were not detected in participants with penetrating thoracic trauma.

### Length of stay

The SA cohort had a mean hospital LOS of 5 (± 4) days which was significantly shorter than that of the Swedish cohort (7 ± 5; p = 0.024). When considering mean LOS in relation to type of injury sustained, no significant differences in duration was found between the blunt injury and penetrating injury groups (blunt: 6 (5) days, PTT: mean 6 (4) days, p = 0.07). Univariate analysis showed that for those with blunt thoracic injury, almost 13% of the variance in LOS is explained by age (R^2^ = 0.129, B coefficient = 0.007, Standard error = 0.001, p < 0.001). None of the other factors entered in the analysis had a significant impact on LOS for those with blunt or penetrating trauma, therefore multivariate analysis was not performed.

### Discharge information

More SA participants were discharged to their homes (99% SA, 56% Sweden, p < 0.001). Thirty-five of the remaining Swedish participants were transferred to another ward/hospital and were lost to follow-up for the purposes of this study. Seven were sent to convalescent care and 32 to follow-up rehabilitation after discharge from the acute care settings. More participants with blunt injury were referred for follow-up care than those with penetrating injury (n = 63 and 3 respectively; p < 0.001).

## Discussion

This prospective observational study is the first to report on levels of pain and shortness of breath experienced by adults with thoracic injury in the first three days of admission, its influence on hospital LOS and patient discharge destination. In addition, it describes and compares the cohort of patients with thoracic trauma in two countries with different socioeconomic levels and the acute care management that they received, mirroring actual clinical practice in the six participating sites.

Males were predominantly involved in thoracic trauma in SA and Sweden, similar to other studies [6, 9]. The SA cohort was younger and were smokers compared to the Swedish cohort that had more participants with chronic respiratory disease. Moreover, the SA participants predominantly had penetrating thoracic injury because of assault, while the Swedish participants had blunt thoracic injury sustained through falls. These findings conform with reports by others [6–9, 16–18]. It contrasts the notion that the majority of thoracic trauma cases is caused by blunt injury [10] and confirms that the types of thoracic trauma cases encountered is determined by the region of the world and socio-economic standing of the community under investigation [4, 5]. The different mechanisms of injury reported in this study reflect the differences in socio-economic levels between the two countries and affect the human body differently. Assault was more often due to stab- or gunshot wounds that did not involve the bony structure of the thorax, although some internal organs of the trunk were injured. This is evident from the higher number of participants in the SA cohort who underwent sternotomy. Falls are more likely to affect the stability of the ribcage and involve injury to extrapulmonary structures. The marked need for rib fixation and additional orthopaedic surgery in the Swedish cohort confirm their higher severity of injury. It may reflect a difference in the management of severe thoracic injuries between the two countries. Rib fixation surgery, although deemed safe and easy to perform within the SA public healthcare sector, remains very expensive and is only reserved for patients with flail injury or severely displaced fractures [19]. None of the SA participants presented with such injuries. Many participants had intrapleural abnormality and the most common was unilateral haemopneumothorax. Pulmonary contusion, lung laceration and diaphragm rupture were less common. Intra-thoracic injuries are common in both blunt and penetrating trauma [10, 13]. Most injuries can be managed with only a chest tube, although not all had an indication for chest tube drainage. Penetrating injuries require intra-thoracic surgery via sternotomy/thoracotomy to a greater extent than blunt trauma. However, blunt trauma with severe injury to the chest wall may require surgery, and the patient is more likely to be operated on if they are managed at a centre where this is provided, such as Sahlgrenska Academy that has a tradition of rib surgery and where 98% of surgeries in this study were performed.

Whole-body CT-scan is standard practice in the management of patients with thoracic trauma in both countries. Additional orthopaedic injury was present in one-third of participants where upper limb fractures and spinal fractures were most common. Liver injury affected a small percentage of participants, but other internal organ injuries were rare. In addition, mild neurological injury affected five percent of participants. This reflects the injury panorama of participants included in the study. Patients with more severe traumatic neurological injury were excluded from this study due to predetermined criteria.

Acute care management of these cohorts were similar for administration of analgesia, MV and high flow nasal cannula oxygen therapy, and few participants were sedated. Some Swedish participants were managed with non-invasive ventilation which may be due to the greater incidence of unilateral and bilateral rib fractures and flail chest injuries. Non-invasive ventilation administered to appropriately selected patients with blunt chest injury decreases complications and the need for intubation and mechanical ventilation [20] but is known to increase LOS [21]. This may be one of the explanations for the longer hospitalisation observed in the Swedish cohort. Other explanations may be the older Swedish blunt injury group, higher incidence of chronic respiratory disease prior to thoracic injury, and socio-economic differences between the two countries where the Swedish state invests more time in planning for home care. In South Africa patients are discharged earlier into the care of their families.

Moderate-to-severe pain was reported by more participants in the blunt injury group during deep breathing in the three days after admission. This observation occurred despite the administration of analgesia. One explanation is that the elderly experience and tolerate pain differently [22]. The Swedish cohort with blunt injury had an average age of 60 years. Hyperalgesia, in the elderly, lasts longer, and combined with slower healing results in prolonged periods of physical discomfort and functional limitations [22]. The effectiveness of analgesia in the elderly is blunted by physiological body system changes and their response to analgesia varies widely which complicates pain management [22].

Most SA and Swedish participants had unilateral haemopneumothorax which was managed with intercostal drainage. The presence of drainage tubes and residual blood in the pleura compress and irritate the intercostal nerves and contribute to inflammation and pain [23]. In addition, pain is caused by damage to myofascial structures [24]. The Swedish cohort had significantly more rib fractures, flail injuries, and sternal fractures and more underwent surgery. Some SA participants had sternotomy due to internal organ injury. Surgery is a common cause of damage to myofascial structures and resultant severe pain. Any movement such as deep breathing and coughing causes tension around the incision site and increases the level of pain experienced [10, 23, 24]. This may relate to the increased pain intensity experienced by both cohorts during such activities.

Shortness of breath was increased more in the blunt injury group when performing any form of activity in the first three days of admission. Trauma causes pain as discussed earlier and leads to reflex dyspnoea. Haemothorax is a recognised cause of shortness of breath [10, 26] and was commonly diagnosed in both cohorts. The Swedish cohort presented with significantly more chronic respiratory diseases. Dyspnoea is a known symptom in chronic respiratory disease [25] and is associated with reduced physical activity [26, 27]. Additionally, dyspnoea and respiratory muscle weakness both influence walking distance in individuals with chronic respiratory disease [27]. This comorbidity could have influenced the blunt injury group’s level of shortness of breath during activity following the acute thoracic trauma sustained.

Thoracic trauma is associated with an increased risk for the development of pulmonary complications such as atelectasis, pneumonia and respiratory distress [10, 13]. Penetrating injury is associated with a low volume of pulmonary contusion and less local inflammation than blunt thoracic injury [28]. Blunt injury was more prevalent in the Swedish cohort that had more diagnosed pulmonary contusion. Atelectasis, pneumonia and respiratory failure with the need for intubation and mechanical ventilation occurred in significantly more participants with blunt injury. The consistently higher levels of pain and shortness of breath experienced from the more complex blunt thoracic injury may explain the higher observed complication rates.

Following the differences between the cohorts, LOS and need of additional hospital care, significantly more Swedish participants were transferred to rehabilitation facilities or convalescent care prior to returning home. The SA cohort was younger, less severely injured and majority were discharged directly to their homes. This may be explained in part by the fact that there are far less rehabilitation facilities in SA for patients recovering from traumatic injury than in high-income countries [29].

Age was associated with longer hospitalisation in the blunt injury group. The complexity of blunt injury could have influenced participants’ ability to optimally ventilate their lungs and cough effectively. Some participants in the Swedish cohort required rib fixation surgery to stabilise their injuries. Post-operative pulmonary complications (PPC) increase duration of hospitalisation [30] and thoracic surgery increases the risk of PPC [30]. Early mobilisation, a strategy that physiotherapists often use as a means of optimising patient ventilation and oxygenation [31], could have been influenced by multiple factors in the current study. The effect of early mobilisation in trauma patients varies as it may decrease duration of mechanical ventilation but its impact on mortality and LOS remains similar to usual care [32].

There were some limitations to this study. No injury severity score data were accessible for direct capturing from patient files in both countries. Retrospective information may be obtained from the Swedish National Trauma Registry; unfortunately, such data cannot be retrieved for the SA cohort, therefore it is not reported. The COVID-19 pandemic may have impacted on the incidence of trauma and hospital admission rates due to various stages of lockdown experienced in SA. Information on the type and dosage of medication used for analgesia and sedation, and the use of epidural and intercostal blockade were not included in the data collection forms and limits extrapolations made about pain management. No data was collected for reasons why intercostal drainage or rib fixation surgery were not performed in cases where it may have been indicated. Data was collected from public healthcare sector university-affiliated hospitals in SA and Sweden which limits the generalisability of results to thoracic trauma management in other settings. A strength of this study is that data capturing was prospective in nature and occurred in parallel to clinical practice. The authors collaborated closely to facilitate an easy and simple data collection process for all involved but there remained some challenges with the amount of data that was captured.

In conclusion, this study confirms the contextual differences in clinical presentation, acute care management and discharge destinations of patients with thoracic trauma in SA and Sweden. It highlights that reassessment of care provided should be prioritised for older patients with blunt thoracic injury to decrease their risk of developing pulmonary complications and prolonged hospital stay.

## Supplementary Information

Below is the link to the electronic supplementary material.

Supplementary file1 (DOCX 22 KB)

## Data Availability

Data supporting the results presented in this manuscript are available from the corresponding author on reasonable request.
