# Supplementary material for: Clinical presentation, acute care management and discharge information of patients with thoracic trauma in South Africa and Sweden: a prospective multicenter observational study
Source: Eur J Trauma Emerg Surg. 2025 Jan 16;51(1):21. doi: 10.1007/s00068-024-02753-y (PMC11739195; doi:10.1007/s00068-024-02753-y)
Supplement: Supplementary file 1 — Supplementary file1 (DOCX 22 KB) [file 68_2024_2753_MOESM1_ESM.docx]

Supplementary table 1: Types and mechanisms of other injuries sustained.

| Variable (n, %) | Description | South African cohort (n=179) | Swedish cohort (n=185) | p-value between cohorts |
| --- | --- | --- | --- | --- |
| Other orthopaedic injuries | Yes | 20 (11) | 90 (49) | <0.001 |
| Types of orthopaedic injuries | None | 162 (91) | 72 (39) | <0.001 |
|  | Soft tissue | 2 (1) | 7 (2) |  |
|  | Upper limb fractures | 9 (5) | 29 (16) |  |
|  | Lower limb fractures | 1 (1) | 0 |  |
|  | Pelvic fractures | 0 | 6 (3) |  |
|  | Facial fractures | 4 (2) | 3 (2) |  |
|  | Spinal fractures | 1 (1) | 22 (12) |  |
|  | Multiple fractures | 0 | 18 (10) |  |
| Injury to internal organs | Yes | 26 (15) | 33 (21) | 0.114 |
| Types of internal organ injuries | None | 153 (86) | 152 (82) | 0.049 |
|  | Heart | 6 (3) | 0 |  |
|  | Kidneys | 2 (1) | 0 |  |
|  | Liver | 9 (5) | 7 (4) |  |
|  | Abdomen | 2 (1) | 0 |  |
|  | Small intestines | 0 | 1 (1) |  |
|  | Large intestines | 1 (0.6) | 1 (1) |  |
|  | Pulmonary | 2 (1) | 3 (2) |  |
|  | Diaphragm | 1 (1) | 0 |  |
|  | Spleen | 3 (2) | 6 (3) |  |
|  | Haematoma | 0 | 2 (1) |  |
|  | Gallbladder | 0 | 1 (1) |  |
|  | Pancreas | 0 | 1 (1) |  |
| Types of internal organ injuries | More than 1 organ injury | 0 | 5 (3) | <0.001 |
| Neurological injury | Yes | 1 (1) | 23 (12) | <0.001 |
| Types of neurological injury | None | 178 (99) | 153 (83) | <0.001 |
|  | Peripheral nerve injury | 0 | 2 (1) |  |
|  | Spinal cord injury | 0 | 10 (5) |  |
|  | Brachial plexus injury | 0 | 1 (1) |  |
|  | Mild TBI | 1 (1) | 18 (10) |  |

Abbreviations: *TBI* traumatic brain injury
